# The Use of Nutraceuticals to Counteract Atherosclerosis: The Role of the Notch Pathway

**DOI:** 10.1155/2019/5470470

**Published:** 2019-05-02

**Authors:** Giorgio Aquila, Luisa Marracino, Valeria Martino, Donato Calabria, Gianluca Campo, Cristiana Caliceti, Paola Rizzo

**Affiliations:** ^1^Department of Medical Sciences, University of Ferrara, Ferrara, Italy; ^2^Department of Chemistry “Giacomo Ciamician, ” Alma Mater Studiorum, University of Bologna, Via Selmi 2, 40126 Bologna, Italy; ^3^Interdepartmental Center of Industrial Research (CIRI) - Energy and Environment, Alma Mater Studiorum, University of Bologna, Bologna, Italy; ^4^Cardiovascular Institute, Azienda Ospedaliero-Universitaria di Ferrara, Cona, Italy; ^5^Laboratory for Technologies of Advanced Therapies (LTTA), University of Ferrara, Ferrara, Italy; ^6^Department of Morphology, Surgery and Experimental Medicine, University of Ferrara, Ferrara, Italy; ^7^Maria Cecilia Hospital, GVM Care & Research, ES Health Science Foundation, Cotignola, Italy

## Abstract

Despite the currently available pharmacotherapies, today, thirty percent of worldwide deaths are due to cardiovascular diseases (CVDs), whose primary cause is atherosclerosis, an inflammatory disorder characterized by the buildup of lipid deposits on the inside of arteries. Multiple cellular signaling pathways have been shown to be involved in the processes underlying atherosclerosis, and evidence has been accumulating for the crucial role of Notch receptors in regulating the functions of the diverse cell types involved in atherosclerosis onset and progression. Several classes of nutraceuticals have potential benefits for the prevention and treatment of atherosclerosis and CVDs, some of which could in part be due to their ability to modulate the Notch pathway. In this review, we summarize the current state of knowledge on the role of Notch in vascular health and its modulation by nutraceuticals for the prevention of atherosclerosis and/or treatment of related CVDs.

## 1. Introduction

“Prevention is better than cure” is the proverb that better describes the impact of eating habits in prophylaxis of many pathologies, including cardiovascular disease (CVD), one of the leading causes of death in industrialized countries [1]. The causes of CVDs are multifactorial and some of these, such as modifiable lifestyle (i.e., tobacco smoking and physical activity) and especially dietary habits, play a major role in this context. In fact, it has been widely demonstrated that a healthy diet, with balanced intake of vegetables, fruits, olive oil, and whole grains, reduces the risk of developing CVDs due to atherosclerosis. Therefore, in the last few years, novel nutritional strategies have been implemented, including dietary modifications and consumption of innovative functional foods and dietary supplements which fall in the category of nutraceutical products. Coined in the end of the ‘90s by Dr. Stephen De Felice, the neologism “nutraceutic” is a combination of the terms “nutrition” and “pharmaceutic” and indicates a discipline that studies the substances that are “a food or part of a food” which can provide “medical or health benefits, including the prevention and treatment of disease” [2]. More recently, nutraceuticals have been defined as “a substance that is cultivated/produced/extracted or synthesized under optimal and reproducible conditions and, when administered orally to normal subjects or patients, would provide the nutrient(s) required for bringing altered body structure and function back to normal, thus improving the health and well-being of the patients” [3]. Consistently, preclinical and clinical studies conducted in the last 20 years have shown an effect of nutraceuticals on prevention and progression of CVDs, specifically on atherosclerosis. Nevertheless, there are studies that did not confirm the protective effect of nutraceuticals, and to shed further light on the action of these compounds, different doses and modality of administration, taking also under consideration the presence/absence of other medications, are being investigated, together with studies of pharmacodynamics and pharmacokinetics. These contrasting results could be also due to the limited number of pathways so far investigated. An example in this context is provided by probiotics, whose supplementation efficacy has given contrasting results. A recently published study shows that probiotic supplements promote endothelial functions in humans with coronary artery disease (CAD) without altering traditional cardiovascular risk factors or the microbiota population but only their transcriptional activity, as indicated by their plasma metabolites profile [4]. Thus, by only looking at the different bacteria population of the microbiota, these effects of probiotics would have been missed. Additionally, by increasing the number of biomarkers of vascular function modified by nutraceuticals, the identification of “super responders” could be achieved, paving the way for “personalized nutraceutical treatments.”

The Notch pathway, originally studied for its role in promoting the survival of cancer cells, is emerging as a key player in the maintenance of the vascular wall health and in the regulation of inflammation [5, 6]. This pathway responds to agents, such as inflammatory cytokines and lipopolysaccharides, whose effects are contrasted by nutraceuticals. The aim of this review is to describe existing studies on the regulation of Notch by nutraceuticals, in order to determine whether the analysis of components of this pathway could represent novel clinical surrogates providing useful information when trying to assess the effect of traditional or emerging nutraceuticals.

## 2. The Steps of Atherosclerosis

Atherosclerosis is a complex multifactorial and multistep chronic inflammatory condition characterized by the progressive accumulation of lipid-loaded fibrous plaques within the artery wall. This pathology, arising from a cumulative damage of the vessel wall, can culminate in atherosclerotic plaque rupture and subsequent thrombus formation [7], leading to its most common clinical manifestations, namely, myocardial infarction (MI) or stroke [8]. The first stage of atherosclerosis development takes place in the endothelium, the intima layer of artery walls. The vascular endothelium is a selective semipermeable continuous monolayer of endothelial cells (ECs) that plays a major role in controlling the vascular tone and vascular wall thickness by synthesizing a plethora of autocrine and paracrine substances, such as nitric oxide (NO), prostacyclin, histamine, prostacyclin, angiotensin II (Ang II), heparin, tissue plasminogen activator (t-PA), and plasminogen activator inhibitor-1 (PAI-I), which affect vascular smooth muscle cell (VSMC) proliferation, leukocyte migration, platelet aggregation, and adhesion [9, 10]. Numerous systemic and hemodynamic risk factors contribute to the onset of endothelial dysfunction (defined as reduced levels of NO, increased endothelium permeability, caused by EC apoptosis and reduction of junctions between ECs, and increased expression of adhesion molecules) which is the first step toward plaque formation [11]. Endothelium discontinuity favors the entry of cholesterol-containing low-density lipoprotein (LDL) particles in the intima of large arteries [10], an event mediated by interaction between LDL apolipoprotein B100 (ApoB100) and matrix proteoglycans. LDL retention predisposes them to oxidative modification [12], and the resulting oxidized LDLs (oxLDL), by binding to a specific receptor, lectin-like oxLDL receptor-1 (LOX1), induce the expression of vascular cell adhesion molecule 1 (VCAM-1) and intercellular adhesion molecule 1 (ICAM-1) by ECs [13, 14]. These adhesion molecules, along with chemotactic molecules, such as monocyte chemoattractant protein-1 (MCP-1) secreted by ECs and VSMCs, mediate the transmigration of monocytes in the subendothelium, where differentiate into macrophages. These later adopt a M1 proinflammatory phenotype and encode a wide range of scavenger receptors (SRs) (i.e., SR-A1 and CD36), useful for facilitating the endocytosis oxLDLs [15]. This oxLDL overload results in acquisition of the “foam cell” phenotype by macrophage and fatty streak formation.

Furthermore, mast cells, T cells, and other inflammation mediators penetrate lesions and, together with foam cells, contribute to the maladaptive proatherosclerotic inflammatory response. Foam cells secrete chemotactic growth factors and metallopeptidases which, through degradation of the extracellular matrix (ECM), support proliferation and migration of VSMCs and leukocytes into the intima [16]. In the intima, the VSMCs produce interstitial collagen and elastin and form a fibrous cap that covers the plaque. This cap overlies the macrophage-derived foam cells, some of which die by apoptosis and release lipids that accumulate extracellularly. This process leads to the formation of a lipid-rich pool called the “necrotic core” of the plaque [7]. The stability of the plaques is influenced by the balance between cap and necrotic core. Stable plaques are characterized by the presence of a small core rich in lipids covered by a thick fibrous cap rich in collagen. Instead, unstable plaques have a thin fibrous cap over a large fatty core [17] and are prone to rupture which leads to the exposure of tissue factors to blood flow and the activation of coagulation cascade with consequent thrombus formation [7, 18]. An intraluminal thrombus in the coronary arteries may decrease blood flow causing ischemic cardiomyopathy: a complete vascular occlusion will cause MI.

## 3. Nutraceutical Supplementation for Atherosclerosis Prevention

Nutraceuticals are classified into (i) dietary supplements: products intended to supplement the diet, which contain one or more dietary ingredients with well-known nutritional functions (i.e., vitamins, minerals, herbs, or herbal active compounds) and (ii) functional foods: consumed as part of a normal diet, they consist in whole foods, along with “fortified, enriched, or enhanced” foods supplemented with known biologically active compounds that, in addition to macro- and micronutrients, provide physiological benefits and are intended for reducing the risk of developing chronic diseases [19]. Some examples of functional foods are garlic, apples, or soybean as remedies for treatment or prevention of a number of diseases, but also vitamin D-fortified milk, useful for counteracting osteoporosis [20]. Lastly, functional food category includes yogurts for their content in probiotics [21], live microorganisms with positive impact on the host through a beneficial action on the intestinal tract, and prebiotics, organic substances, found in several vegetables and fruits, which selectively enhance the activity of some groups of bacteria [22].

This traditional definition of nutraceutical classes intended that both nutraceutical ingredients and functional foods derived preferentially from food products. However, the hodiern nutraceutical includes also non-food-derived active metabolites, which are safe and useful as novel sources for modern nutraceuticals and functional foods, such as medicinal plant-derived compounds [23], marine bioactive compounds [22], and amino acids derived from bacteria fermentation [24].

**Figure 1:**
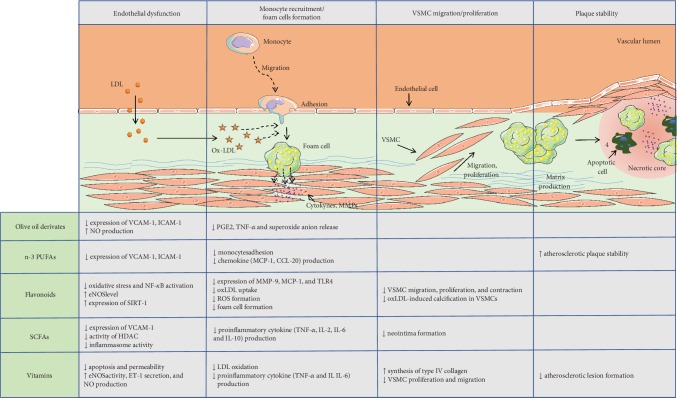
Beneficial effects of major nutraceuticals on atherogenesis key steps. Highlights of the main findings of *in vitro* and *in vivo* studies which have investigated the mechanisms underlying the benefits of the major nutraceuticals (olive oil derivates, n-3 PUFAs, flavonoids, SCFAs, and vitamins) at different stages of atherosclerosis development, including endothelial dysfunction, monocyte recruitment, foam cell formation, VSMC migration and proliferation, and plaque stability.

Several classes of nutraceuticals have been shown to have potential benefits in the treatment of atherosclerosis (reviewed in [25]) (Figure 1) and CVDs, and the ones for which strong evidence exists for atherosclerosis protection are described below and summarized in Table 1.

### 3.1. Olive Oil Derivates

Nutritional intake of olive oil, a key component of Mediterranean diet, has been associated with the prevention of CVDs, thanks to its content in monounsaturated fatty acids [26]. The 75% of fatty acids in olive oil is represented by monounsaturated oleic acid which counteracts endothelial dysfunction and reduces the tumor necrosis factor-alpha- (TNF-*α*-) induced apoptosis in VSMCs [27]. Olive oil is also a source of diverse phenolic compounds, such as hydroxytyrosol, oleocanthal, oleuropein, lignans, and pinoresinol, and numerous experimental, clinical, and epidemiological investigations support the beneficial properties of these olive derivates in CVDs [28, 29]. For instance, as for oleic acid, hydroxytyrosol reduces the expression of cell surface adhesion molecules (VCAM-1, ICAM-1, and E-selectin) in human umbilical vein endothelial cell line (HUVEC)[30]. Furthermore, hydroxytyrosol, as well as its derivates, inhibits the release of superoxide anions, prostaglandin E2 (PGE2), and TNF-*α* and the expression of cyclooxygenase 2 (COX2) in human monocytes [31]. Similarly, peracetylated hydroxytyrosol, a hydroxytyrosol derivate, attenuates lipopolysaccharide- (LPS-) induced proinflammatory cytokine production in murine peritoneal macrophages [32]. Interestingly, a diet rich in polyphenol powder, obtained from olive mill wastewater, enhanced antioxidant mechanisms and reduced oxidative stress-induced damage in chickens [33]. Moreover, *in vitro* treatment with hydroxytyrosol, or polyphenol extracts, from extra virgin olive oil, protects against the endothelial dysfunction induced by hyperglycemia and free fatty acids through modulation of NO production and endothelin-1 (ET-1) expression [34]. These observations have been confirmed by the NUTRAOLEUM study, a randomized double-blind controlled trial, which supported the hypothesis of beneficial effects of virgin olive oils on biomarkers of endothelial dysfunction in healthy adults. This study demonstrated that administration of olive oil enriched in phenols, especially in hydroxytyrosol, other than improving plasma high-density lipoprotein (HDL) levels, ameliorates the systemic ET-1 levels [35]. These data mirror those obtained in two other clinical trials which tested the effect of polyphenol-enriched olive oils on HDL- and endothelial-related markers in hypercholesterolemic subjects. In these studies, these functional olive oils promoted cardioprotective effects, as indicated by increased levels of fat-soluble antioxidants and antioxidant enzymes, improved HDL subclass distribution, reduced the level of DNA oxidation, and ameliorated endothelial function [36, 37]. Very recently, it has been also demonstrated that hydroxytyrosol blunts endothelial dysfunction by ameliorating mitochondrial function and reducing mitochondrial oxidative stress [38], suggesting a potential mitochondria-targeting antioxidant activity of hydroxytyrosol in the inflamed endothelium.

### 3.2. N-3 Polyunsaturated Fatty Acids

Polyunsaturated fatty acids (PUFAs) are fatty acids that contain two or more bonds in their carbon chains. Depending on the position of the carbon-carbon double bond closest to the omega (methyl) end of the molecule, PUFAs can be classified in omega-6 and omega-3 series of fatty acids which, other than being vital constituents of cell membranes, constitute the substrates for synthesis of eicosanoids (i.e., prostaglandins, prostacyclins, thromboxanes, and leukotrienes), mediators of inflammatory response and regulators of blood pressure and coagulation [39]. In the end of the 80's, an examination of the composition of Eskimos' diet revealed an association between the low mortality rate due to cardiovascular events in this ethnic group and their diet rich in PUFAs derived from sea fish [40]. This observation prompted the researchers to investigate the biological functions of these compounds and their role in human health and pathology prevention. During the past 20 years, the research on the role of omega-3 PUFAs in CVD has flourished, with the majority of scientific research being focused on eicosapentaenoic acid (EPA) and docosahexaenoic acid (DHA), both displaying beneficial cardiovascular effects. In fact, behind the possible cardioprotective effect of these omega-3 fatty acids, other than the reduction of triglyceride (TG) levels [39], additional modes of actions have been proposed, which include hypotensive effect [41], thrombosis reduction [42], and also decrease in malignant arrhythmias [43]. Moreover, numerous *in vitro* and *in vivo* investigations have shown that omega-3 PUFAs are able to modulate diverse key steps involved in atherosclerotic plaque formation (reviewed in [44]). Specifically, omega-3 PUFAs exert potent anti-inflammatory properties in polarized macrophages [45, 46] and are able to inhibit the expression of adhesion molecules by ECs [39, 47], thus decreasing leukocyte infiltration into the vascular wall [48]. In ApoE^−/−^ mice, omega-3 PUFA treatments significantly attenuated the development and destabilization of atherosclerotic plaques [49]. In diabetic mice, supplementation with *ω*-3 fatty acids extracted from microalgae decreased the percentage of T lymphocyte CD4^+^-producing proinflammatory cytokines [45].

Several clinical trials have been conducted to assess the cardiovascular benefits of EPA and DHA treatments (i.e., diet and reinfarction trial (DART) [50], Japan EPA Liquid Intervention Study (JELIS) [51], and Gruppo Italiano per lo Studio della Sopravvivenza nell'infarto (GISSI) [52]). In 2002, a meta-analysis of eleven randomized clinical trials revealed that *ω*-3 PUFA supplementation reduced overall mortality, mortality due to myocardial infarction, and sudden death in patients with coronary heart disease (CHD) [53]. In the same year, the American Heart Association (AHA) recommended doses of total EPA and DHA between 2 and 4 g/day, to achieve a TG level reduction by 25-30% in normal and hyperlipidemic individuals [54]. After the publication of these guidelines, a large number of observational studies on omega-3 PUFA intake and CHD risk were conducted. In 2017, in an updated scientific advisory of AHA, the authors confirmed omega-3 fish oil supplements, in consultation with a physician, as a secondary—and not primary—prevention of sudden cardiac death in patients with prevalent CHD and in patients with heart failure [55]. In contrast, a recent meta-analysis of 10 trials involving 77917 individuals did not provide support for the AHA recommendations in individuals with a history of CHD for the prevention of fatal CHD, nonfatal MI, or any other vascular events [56]. Consistently, in a recent clinical trial, three months of treatment with PUFAs at a dose of 2 g/die did not improve endothelial function in patients with type 2 diabetes and high cardiovascular risk [57]. Similarly, in the ASCEND study, after a follow-up of 7.4 years, patients with diabetes and no evidence of cardiovascular disease, who received a daily supplement of *ω*-3 fatty acids, did not show a significantly lower incidence of serious vascular events than those who received placebo [58]. Despite the evidence from these last randomized trials that omega-3 has little or no effect on cardiovascular outcomes, clinical guidelines still recommend the use of omega-3 fatty acid supplements for the secondary prevention of CHD [55] and consider the beneficial effect of consuming fish and seafood, for their content in omega-3 PUFAs [59]. To date omega-3 PUFA effects on cardiovascular endpoints remain still unclear and might vary based on different types/doses of dietary omega-3 intakes and the presence of other medications (i.e., statins) in the clinical trials performed so far. Consistently, the recent results of the REDUCE-IT trial, which involved 19212 patients with elevated triglyceride levels at risk for ischemic events, showed that treatment with 4 g/die of EPA, a dose twice and 4-times higher compared to the dose tested in the Omega-FMD study [57] and ASCEND study [58], respectively, resulted in decreased risk of primary and secondary composite cardiovascular end points (i.e., cardiovascular death, nonfatal myocardial infarction, nonfatal stroke, coronary revascularization, or unstable angina) of 25 and 26%, respectively [60]. Several ongoing large randomized clinical trials (i.e., EVAPORATE [61], VITamin D and OmegA-3 TriaL, VITAL [62], STatin Residual Risk Reduction With EpaNova in HiGh CV Risk PatienTs With Hypertriglyceridemia, and STRENGTH (ClinicalTrials.gov Identifier NCT02104817)) will shed more light on the possible associations between omega-3 supplementation and reduction of risk of major cardiovascular events.

### 3.3. Flavonoids

Flavonoids, antioxidants present in fruit and vegetables, represent the most abundant polyphenolic constituents in foods of plant origin [63]. It has been estimated that the daily dietary intake of flavonoids is at least 1 gram per person and thus it is higher than that of all other known dietary antioxidants (e.g., vitamins C and E intake from food are estimated less than 100 mg/day) [64, 65]. Flavonoids present a 15-carbon (C6-C3-C6) skeleton, which generally consists of two phenyl rings and a heterocyclic ring. Approximately 8000 flavonoids have been identified based on C3 structure variations and degree of oxidation, and these include flavonols, flavones, isoflavones, flavanones, flavan-3-ols, and anthocyanidins [66]. In the context of atherosclerosis, flavonoids exert various beneficial effects, such as improvement of endothelial function and reduced oxidative stress and inflammation, and also antiplatelet, antihypertensive, and vasodilatory actions, inhibition of cholesterol synthesis, alteration of HDL function, and increased insulin sensitivity (reviewed in [67–69]). Flavanols are the subclass of flavonoids [70, 71] which have attracted most of the attention in the cardiovascular research community, since numerous epidemiological and mechanistic studies have supported the role of flavanols, particularly catechins, contained in cocoa and green tea, in counteracting endothelial dysfunction and atherosclerosis development [63, 72–76]. The mechanisms underlying the cardiovascular protective effects of catechins may include the inhibition of endothelial cell apoptosis by decreasing oxidative stress and ameliorating mitochondrial injury [77] and attenuation of proliferation of VSMCs by regulating anti-inflammatory and antioxidative enzyme heme oxygenase-1 (HO-1) [78]. In macrophage cell lines, catechins also mediate suppression of expression of metalloproteinase-9 (MMP-9), monocyte chemoattractant protein-1 MCP-1, and Toll-like receptor 4 (TLR4), which have a major role in atherosclerosis [79]. Moreover, in macrophages, epigallocatechin-3-gallate (EGCG) suppresses oxLDL uptake and foam cell formation [80]. In ApoE^−/−^ mice, catechin consumption has been associated with reduced susceptibility of LDL to oxidation and aggregation and thus to the reduced atherosclerotic lesion area [81]. Recently, *in vivo* findings show that catechins reduce circulating LDL cholesterol and protect HUVECs against oxidative injury and decrease arterial vasoconstriction through reduction of H_2_O_2_ activity and eNOS level restoration [82, 83].

Similarly to flavanols, flavonols, and primarily quercetin, reduce atherosclerosis lesion area *in vivo* by affecting the oxidative stress status [84, 85]. The mechanism underlying this protection, which resembles those of flavanols, may involve attenuation of endothelial dysfunction [86], induction of HO-1 [87] and sirtuin-1 (SIRT-1) [88], and reduction of NF-*κ*B activity [89]. It has been also suggested that quercetin may also inhibit VSMC migration, proliferation, and contraction [90, 91] and oxLDL-induced calcification in VSMCs, by targeting the ROS/TLR4 signaling pathway [92]. Recently, it has been also demonstrated that quercetin inhibits ox-LDL-induced ROS formation in mouse peritoneal macrophages by limiting the activation of NADPH-oxidase, which in turn may lead to the observed attenuation of high-fat diet-induced atherosclerosis in ApoE^−/−^ mice [93, 94]. Additionally, quercetin reduces ROS levels in aortas of hypertensive rats by interfering with MMP-2 activity, thus limiting vascular remodeling [95]. Similarly, quercetin metabolites, like quercetin-3-glucuronide, generally found in onions, broccoli, and apples, have been shown to exhibit antioxidant, anti-inflammatory, and also antihyperglycemic properties both *in vitro* and *in vivo* [96–98].

The Zutphen Elderly Study was the first investigation in 805 men (aged 65-84 years, followed up for 5 years) which assessed the inverse correlation between flavonoid intake from tea, with a high content in quercetin, and the mortality from CHD, together with the incidence of MI [99]. Such correlation was confirmed by the Rotterdam Study, in which it was found that increased intake of tea and flavonoids may contribute to the primary prevention of ischemic heart disease [100]. Conversely, the protective effect of consumption of quercetin-rich tea, against ischemic heart disease, was not confirmed in other studies, such as the Caerphilly Study of Welsh men [101] and the Health Professionals Follow-Up Study [102]. Recently, the FLAVO study, a randomized, double-blind, placebo-controlled crossover trial performed in 37 (pre)hypertensive men and women (40-80 years), compared the cardioprotective effects of quercetin and epicatechin supplementation, both exerting similar *in vitro* and *in vivo* atheroprotective functions. The study has shown that, unlike quercetin-3-glucoside, supplementation of pure epicatechin improves endothelial function and reduces inflammation, thus reducing CVD risk factors [103].

Together, these data suggest that flavonoids, especially quercetin and catechins, might exert cardiovascular health benefits but larger trials are needed to draw conclusive evidence for the cardiovascular protective effects of these natural antioxidants, in the presence and absence of commonly used therapies for CVD.

### 3.4. Short-Chain Fatty Acids

In 1954, Walker and Ardvidsson [104] and Higginson and Pepler [105] were the first to suggest that low plasma lipid levels and the absence of atherosclerosis in primitive African populations were mostly due to their fiber-rich diet. In the 70's, this hypothesis was formalized by Burkitt et al. [106] and Trowell [107], proposing that the interaction among the dietary components can disturb the course of atherosclerosis onset, pointing out at the pivotal role played by fibers in modulating the serum lipid level. Since then, diverse epidemiological studies have reported an inverse relationship between fiber consumption and cardiovascular risk and that the beneficial effects of fibers would primarily reside in an improvement of the serum lipid profile (reduced total serum and LDL cholesterol levels) and reduction in blood pressure and systemic inflammation. In a recent umbrella review of all published meta-analyses (from January 1, 1980 to January 31, 2017), it emerged that dietary fibers can reduce chances of developing CHD and stroke between 7% to 24% and the overall morbidity and mortality caused by cardiovascular disease from 17% to 28% [108]. These observations mirror those described in another systematic meta-analysis review, performed in 2014, which investigated the association of fiber consumption and all-cause and cause-specific mortality in 42 cohorts (from 25 studies). This analysis showed a 23% reduction of the mortality rate for CVDs and a concomitant 17% decrease for cancer-related death and a 23% reduction for all-cause mortalities [109]. Based on these scientific evidences, the Food and Drug Administration (FDA) strongly recommends consumption of fiber-rich food for health promotion and disease prevention, suggesting that an adequate intake of dietary fibers should be at least 25 grams/day for a 2000-calorie diet (https://www.accessdata.fda.gov/scripts/InteractiveNutritionFactsLabel/factsheets/Dietary_Fiber.pdf).

The beneficial effects on lowering total serum cholesterol are attributed to three major mechanisms: (i) prevention of bile salt reabsorption from the small intestine which leads to increased fecal excretion of bile acids, (ii) reduced glycemic index and insulin resistance, which can result in the inhibition of hydroxymethylglutaryl coenzyme A (HMG-CoA) reductase activity and hepatic cholesterol synthesis [110, 111], and (iii) production, from anaerobic bacterial fermentation of undigestible fibers, of short chain fatty acids (SCFAs), mainly acetate, propionate, and butyrate [112]. SCFAs inhibit the production of proinflammatory cytokines and the recruitment of immune cells to ECs, mechanisms mediated by binding to the free fatty acid (FFA) receptor types 2 and 3 and G-protein-coupled receptor 109A (GPR109A) and by inhibiting intracellular activity of histone deacetylases (HDACs), enzymes which can regulate multiple molecular processes involved in atherogenesis [97, 113–115]. Recently, it has emerged that SCFAs inhibit LPS- and TNF-*α*-induced endothelial expression of VCAM-1, through activation of GPR41/43, which are SCFA receptors expressed in ECs, and inhibition of HDAC activity [116, 117]. The anti-inflammatory activity of SCFAs has been also evaluated *in vivo*: in a partially ligated carotid artery (PLCA) mouse model of atherosclerosis, it has been found that butyrate repressed endothelial Nlrp3 (Nlr family pyrin domain-containing 3) inflammasome activation in ECs, via redox signaling pathways, and prevented arterial neointima formation. On the contrary, acetate and propionate exerted minimal inhibitory effects on inflammasome activation and even seem to augment the arterial neointima formation in the PLCA model [117]. The antiatherosclerotic activity of SCFAs was also confirmed by a recent study by Chen and collaborators showing that SCFAs from pectin fermentation are able to inhibit intestinal cholesterol absorption, to decrease serum total and low-density lipoprotein cholesterol and to protect ApoE^−/−^ against diet-induced atherosclerosis [118]. Also, in this case, the authors showed that the beneficial effects of SCFAs are mediated only by butyrate through a mechanism which involves increased expression, in the small intestinal mucosa, of ATP-binding cassette (ABC) transporters G5 (*Abcg5*) and G8 (*Abcg8*), which limit intestinal absorption and facilitate biliary secretion of cholesterol [118, 119].

### 3.5. Vitamins

An adequate intake of vitamins, from food or dietary supplements, is commonly considered as indispensable for maintenance of good health, and thus, since their discovery in the early 1900s, vitamins have been considered the most promising nutraceuticals for the prevention of diverse pathologies, including atherosclerosis-derived CVDs. In this context, vitamins C and E have received the most attention since, according to the “oxidation hypothesis” of atherosclerosis onset [120, 121], antioxidant organic compounds, like these two vitamins, could represent the first line of defense against LDL oxidation, the first step in the formation of atherosclerotic plaques. Moreover, during the last two decades, other antiatherogenic mechanisms of action have been ascribed to vitamins C and E (reviewed in [25, 122, 123]), such as stimulation of endothelial cell proliferation, thanks to their ability to increase the synthesis of type IV collagen, reduction of apoptosis induced by high glucose conditions, LPS or TNF-*α* [122, 123], enhancement of eNOS activity by stabilization of the eNOS cofactor tetrahydrobiopterin (BH4) [124], and tightening of the permeability barrier of ECs [124]. Similarly, for vitamin E, the antiatherosclerotic action has been found, which is independent from its antioxidant properties, such as reduced cholesterol synthesis by inhibiting HMG-CoA reductase, increased eNOS activity, reduced NF-*κ*B-dependent synthesis of ICAM-1 and VCAM-1, reduced platelet aggregation, and inhibition of VSMC proliferation [123, 125].

These *in vitro* findings were confirmed by studies in animal models of atherosclerosis (as reviewed in [126]). For instance, vitamin E, in an age-dependent manner, is able to inhibit atherosclerosis in ApoE^−/−^ mice by decreasing serum oxLDL and vasculature mRNA expression of genes involved in cholesterol transportation [127]. Similarly, *α*-tocopheryl phosphate (a natural form of vitamin E) limits atherosclerosis lesions in hypercholesterolemic rabbits [128] and in ApoE^−/−^ mice by limiting aortic superoxide formation and by reducing circulating plasma levels of proinflammatory markers [129], Similarly, vitamin E deficiency, caused by disruption of the *α*-tocopherol transfer protein gene, increased the severity of atherosclerotic lesions [130].

Likewise, vitamin C limits endothelial dysfunction in animal models of atherosclerosis: long-term supplementation (26/28 weeks) of vitamin C restored eNOS activity in the aorta of ApoE^−/−^ mice [131], whereas chronic hypoascorbemia has been associated to an elevated lipoprotein(a) (Lp(a)) accumulation in the vasculature and increased atherosclerotic lesion development in gulonolactone-oxidase-deficient (Gulo^−/−^)/Lp(a)^+^ mice, a model lacking of endogenous ascorbate synthesis and expressing human Lp(a) [132].

*In vivo* studies have investigated the antiatherosclerotic effect of a combined vitamin C and vitamin E supplementation in mouse models of this pathology: vitamin C/vitamin E cocktail inhibited the development of fatty streak lesions in the LDLr^−/−^ mice [133] and limited aortic *Vegf* and *Vegfr-2* expression in ApoE^−/−^ mice [134] compared to nontreated littermates. In a very recent study, in atherogenic diet-fed (scavenger receptor class B type 1) SR-B1 KO/ApoER61^h/h^ mice, a murine model of dyslipidemia, progressive atherosclerosis, CHD and premature ischemic death, combined with administration of vitamins C and E reduced serum total cholesterol and triglyceride levels, improved HDL antioxidant function, and lowered serum TNF-*α* levels [135].

Other than vitamins C and E, vitamins A and D show potential antiatherosclerotic properties (as reviewed by [136]). The wide range of vitamin D beneficial functions includes reduction of endothelial dysfunction and VSMC proliferation and migration, as well as downregulation of the atherosclerosis-related inflammatory and immune processes [136]. Less is known about vitamin A (retinol) in this context: retinoic acid (RA) metabolites, derivatives of vitamin A, limit VSMC growth, differentiation, and proliferation [137] and prevent high-fat diet- (HFD-) induced atherogenesis in ApoE^−/−^ mice via the upregulation of aforementioned transporters ABC-A1 and ABC-G1 [138]. Peculiarly, in the same animal model, vitamin A deficiency stimulates atherogenesis, prevented by *β*-carotene supplementation [139]. All-trans retinoic acid (ATRA), a derivative of vitamin A, has been recently shown to reduce the plaque size in a rabbit model of HFD-induced atherosclerosis by inhibiting platelet aggregation, by decreasing caveolin-1 expression and ET-1 secretion and by enhancing eNOS activity and NO formation [140–143].

Despite the promising *in vitro* and *in vivo* findings supporting the antiatherosclerotic properties of vitamins and their metabolites, at the clinical level, the results obtained have been contradictory: albeit the vast majority of the literature have correlated a low level of the above described vitamins with early atherosclerosis onset, major risk of a CVD events, and heart failure in human [144–152], it is undeniable that the various clinical trials, performed so far, lack consistency. For instance, a few studies have evidenced that supplementation of vitamins C and E, alone or in combination, may delay the progression of atherosclerosis [153, 154] and reduce cardiovascular mortality in healthy women [155], whereas many other trials, like the MRC/BHF Heart Protection Study Heart Protection Study Collaborative Group [156], GISSI-Prevenzione [52], VEAPS [157], HOPE [158], and Supplementation en Vitamines et Mineraux Antioxydants (SU.VI.MAX) [159], reported that these vitamins do not produce any significant difference in the incidence of cardiovascular events and CVD-related mortality. Contextually, in 2011, a systematic review of 51 trials showed no significant reduction in mortality and cardiovascular risk associated with vitamin D supplementation [160], having conclusions mirroring those obtained from vitamin C-centered meta-analysis performed in 2016 [161]. It is expected that new insights will arise from the ongoing randomized, double-blind, placebo-controlled VITAL trial, which will evaluate the long-term effects of high-dose vitamin D supplementation on CVD events in 25874 U.S. adults [62].

Only a few trials have been performed to assess the effects of vitamin A on CVDs. In 1996, the beta-carotene and retinol efficacy trial (CARET) tested the effect of beta carotene and vitamin A supplementation on the incidence of lung cancer and cardiovascular death in 18314 high-risk participants, specifically smokers and workers exposed to asbestos [162]. This trial was stopped because participants randomly assigned to vitamin supplementation, compared to the placebo group, exhibited a 28% increase in incidence of lung cancer and a 17% increase of overall mortality rate and a higher rate (26%) of cardiovascular disease mortality, which decreased during the 6-year follow-up after the vitamin integration was stopped [162, 163].

A recent meta-analysis of 18 studies, which involved a total of 2 million participants, concluded that taking multivitamins does not prevent heart attacks, strokes, or cardiovascular death, even though it seems to be associated with a lower risk of CHD incidence [164]. In the absence of studies showing their efficacy in primary prevention of CVDs, the AHA does not recommend vitamin supplementation for healthy subjects, and similarly, the U.S. Preventive Services Task Force (USPSTF) states that the current evidence is insufficient (I) to assess the balance of benefits and harms of the use of multivitamins for the prevention of cardiovascular disease or cancer (I statement). The USPSTF also discourages (D) the use of *β*-carotene or vitamin E supplements for the prevention of cardiovascular disease or cancer (D recommendation) [165].

### 3.6. Other Emerging Antiatherogenic Nutraceuticals

#### 3.6.1. Berberine

Berberine (BBR), a quaternary ammonium salt from the protoberberine group of isoquinoline alkaloids (5,6-dihydrodibenzoquinolizinium derivative) found in *Berberis* species plants (Berberidaceae), exhibits many different types of biological activities, including its effectiveness in lipid disorders and hyperglycemia [166]. The poor intestinal absorption and bioavailability of BBR are the main drawback when orally administered even though its metabolites maintain higher concentration in plasma, behaving like the pharmacologically active forms of BBR; its main metabolite berberrubine (M1) tautomerizes to a highly conjugated, electroneutral quinoid structure [167] reaching a high plasma concentration as a consequence of a more efficient intestinal absorption [167].

Several preclinical studies as well as clinical trials suggest a beneficial role of BBR in endothelial dysfunction and dyslipidemia [166]; in ECs, BBR attenuates LDL oxidation induced by ROS and reduces apoptosis modulation, chromosome condensation, cytochrome c release, and caspase-3 activation. It has also been reported that BBR reversed NOX4-derived ROS production in HUVECs, at least in part due to the regulation of adenosine monophosphate-activated protein kinase (AMPK) activation. In both cultured endothelial cells and blood vessels isolated from rat aorta, BBR enhanced eNOS and promoted a glutathione peroxidase (GSH-Px) and superoxide dismutase (SOD) hyperactivation in the liver of mice, attenuating H_2_O_2_-induced ROS [168]. BBR elevates LDL receptor (LDLR) expression in human liver cells through inhibition of proprotein convertase subtilisin/kexin type 9 (PCSK9) transcription, an enzyme that posttranscriptionally upregulates LDLR [169]. In rat liver, a combination of BBR with simvastatin increased the LDLR gene expression to a level significantly higher in comparison to monotherapies [166]. In human macrophage-derived foam cells treated with oxLDL, BBR inhibits the expression of LOX-1 as well as the oxLDL uptake by macrophages and reduces foam cell formation in a dose-dependent manner by activating the AMPK-SIRT1-PPAR*γ* pathway [170]. We recently reported that BBR prevents the oxLDL- and TNF-*α*-induced LOX1 expression and oxidative stress in HUVECs, key events leading to NOX, MAPK/Erk1/2, and NF-*κ*B activation linked to endothelial dysfunction [171], and consistently, Abidi and colleagues had previously shown, both *in vitro* and *in vivo*, that BBR reduces VCAM-1 expression induced by LPS [172]. We recently observed that M1 inhibited intracellular xanthine oxidase activity, one of the major sources of ROS in vasculature, and reduced the expression ICAM-1 [173]. The lipid-lowering activity of BBR, alone or in association with other nutraceuticals, has been clearly confirmed in a relatively large number of randomized clinical trials which support the safety of a short-term use of this nutraceutical, especially when used at a lipid-lowering dose [166].

#### 3.6.2. Carotenoids

Carotenoids represent a group of pigments widely distributed in nature. They contribute to the red, orange, and yellow colors found in many flowers, fruits, and vegetables, where they act as photoprotectors and as attractant for insects and animals for pollination and seed dispersal. Since animals and humans are unable to synthesize carotenoids de novo, carotenoids are essential nutrients and important health beneficial compounds [174, 175]. Carotenoid consumption improves the metabolic profile, decreasing the incidence of diabetes, lowers LDL levels, and improves blood pressure by ameliorating the bioavailability of NO [175]. Apart from the well-established function of carotenoids as provitamin A, some carotenoids, such as lycopene and astaxanthin, are strong antioxidants and have a protective function in reducing the risk of both cancer and cardiovascular diseases [175, 176]. Xanthophylls, such as lutein and zeaxanthin, are essential components of the macular pigments in the eyes and offer protection against macular degeneration, the leading cause of age-related blindness [175, 177, 178]. Lutein exhibits strong antioxidant properties *in vitro* and *in vivo* [178], and low serum levels of lutein have been associated both with increased values of common carotid intimal medial thickness (CCA-IMT) and myocardial infarction [179]. An 8-year follow-up study, performed on 43.738 men with no history of cardiovascular disease or diabetes, showed a significant inverse correlation between lutein intake and risk for ischemic stroke [179]. A case control study found that the risk for MI was inversely correlated with adipose tissue lutein content and inversely proportional to dietary lutein intake [178].

Due to their antioxidant activity, lycopene, lutein, zeaxanthin, and astaxanthin are able to attenuate the atherosclerotic process. Lycopene, a fat-soluble carotenoid without provitamin A activity, is the pigment responsible for the distinctive red color in tomatoes and watermelon, and it is a powerful antioxidant and free radical quencher [180]. High plasma lycopene levels have been associated with reduction in aortic stiffness in patients with metabolic syndrome [181]. Conversely, low plasma levels of lycopene were associated with increased risk of atherosclerotic lesions and with an increased risk of acute coronary events or stroke [179]. Similarly, in a case control study performed in patients suffering from heart failure (NYHA class II-III), the left ventricular ejection fraction was significantly and positively correlated with plasma lycopene levels: NYHA class II patients showed significantly higher levels of lycopene than class III patients [179]. High lycopene consumption has been associated with a decreased risk of CVD, including atherosclerosis, myocardial infarction, and stroke. In a study performed on healthy male volunteers, lycopene supplementation improved the endothelial function, together with a significant decrease in serum levels of CRP, ICAM-1, and VCAM-1 and an improvement in the atherosclerotic risk factors (lipid profile and systolic blood pressure level) [181]. A meta-analysis using a random effects model of all studies between 1955 and September 2010 investigating the effect of lycopene on blood lipids or blood pressure for a minimum duration of 2 weeks suggests that lycopene taken in doses ≥ 25 mg daily is effective in reducing LDL cholesterol by about 10% which is comparable to the effect of low doses of statins in patients with slightly elevated cholesterol levels [180].

Ketocarotenoid astaxanthin is the main carotenoid present in aquatic animals (salmon, trout, red seabream, shrimp, lobster, and fish eggs), contributing to the pinkish-red color of their flesh, and also in some birds (flamingoes and quails in particular) [179]. Astaxanthin exhibits a free radical-quenching potency that is, on an equimolar basis, double than the potency of *β*-carotene [178], about 100-fold greater than the antioxidant potency of *α*-tocopherol [178], and approximately 6000 times the potency of ascorbic acid [178]. Astaxanthin demonstrated to exert beneficial effects on the heart, both by reducing inflammation and by modifying blood levels of LDL-C and HDL-C; moreover, it reduces macrophage infiltration and apoptosis in vascular lesions, thus improving plaque stability by increasing adiponectin [179]. Astaxanthin inhibits also the production of oxLDL [178] and their uptake by activated intravascular macrophages [178] and inhibits the release of atherogenic ROS, NO, and proinflammatory cytokines [178]. 8 weeks of a dietary supplementation with 2 mg of astaxanthin daily by a group of healthy postmenopausal women produced a significantly greater increase in total plasma antioxidant machinery than what was elicited by placebo and a significantly greater decrease in the plasma concentration of thiobarbituric acid-reactive substances (the mixed reaction products of nonenzymatic oxidative lipid peroxidation) [178]. Dietary astaxanthin also contributes to healthy blood flow through the vasculature by promoting aortic and coronary artery vasodilation and increases the flexibility of red blood cell membranes (with an acceleration of red blood cell flow through the blood vessels) [178].

#### 3.6.3. Red Yeast Rice

Red yeast rice (RYR) is a Chinese herbal supplement produced by fermenting white rice with the yeast, *Monascus purpureus*, used to flavour, color, and preserve foods and as a traditional medicine for many years. RYR contains a variety of monacolins, which inhibit HMG-CoA reductase, the rate-limiting step in cholesterol synthesis. Approximately 90% of the total monacolin content of RYR consists of monacolin K, chemically identical to lovastatin, and its hydroxy acid form, monacolin KA [182]. Other active ingredients with the potential to lower cholesterol in commercially available RYR products include plant sterols (*β*-sitosterol, campesterol, and stigmasterol), isoflavones, and monounsaturated fatty acids [183]. The first prospective, double-blind, placebo-controlled study evaluating RYR in an American population was conducted by Heber et al. in 1999 in eighty-three healthy adults with untreated hyperlipidemia that followed the AHA cardioprotective diet (less than 10% of calories from saturated fat and less than 300 mg from cholesterol per day). They were randomly assigned to receive 2.4 g per day of RYR or placebo, for 12 weeks. Compared to baseline, LDL-C levels decreased by 22% in the RYR-treated group [184]. Other clinical trials have found that a relatively small dose of RYR (equivalent to a daily lovastatin dose of 5 to 7 mg) is as effective as 20 to 40 mg of pure lovastatin in lowering cholesterol [185]. Becker et al. compared the efficacy of an alternative treatment composed by RYR, fish oil, and therapeutic lifestyle changes with simvastatin 40 mg per day in 74 primary prevention patients with known or newly diagnosed hypercholesterolemia [186]. Depending on baseline LDL-C, patients took 1200 mg of RYR (10 mg lovastatin) or 1800 mg (15 mg lovastatin) twice a day, for 12 weeks. At the end of the study, both groups had a similar reduction in LDL-C and no significant differences were found between the two groups. Interestingly, participants in the RYR-fish oil and life style change group lost more weight during the study (−4.7 ± 2.4 kg vs −0.3 ± 2.2 kg) and had a significant reduction in triglycerides compared with the simvastatin group. Li et al. published a large meta-analysis, which examined the effectiveness and safety of RYR as an alternative approach for treating dyslipidemia. Thirteen randomized, placebo-controlled trials were included (from 1999 to 2013) with treatment duration of 4 weeks to 6 months and no serious side effects were reported. Overall, RYR significantly lowered total and LDL-C levels (*P* < 0.001) compared with placebo and this effect did not appear to be related to the dose, duration of therapy, or geographic location [187]. In another small observational study, 25 dyslipidemic patients with a history of intolerance to lipid-lowering medications were treated with RYR for more than 4 weeks. In accordance with other studies, RYR significantly lowered LDL-C by 21% in this clinical population during the period of treatment [188]. The China coronary secondary prevention study (CCSPS) so far is the only randomized, double-blinded, placebo-controlled, multicentered study demonstrating that monacolin K reduces cardiovascular risk [189]. This trial recruited 4870 Chinese patients with a history of MI and moderate hypercholesterolemia. Patients were randomized to receive twice-daily treatment of a capsule of Xuezhikang (XZK), containing 2.5 to 3.2 mg of monacolin K equivalent to a total daily lovastatin dose of 10 to 12.8 mg or placebo. After 4.5 years, XZK was associated with a highly significant reduction in frequency of coronary events (10.4% in the placebo vs 5.7% in the XZK group) and a relative risk reduction of 45% [189]. Treatment with XZK also significantly decreased total mortality by 33%, cardiovascular deaths by 30%, and the need for coronary revascularization by 33%. Total cholesterol and LDL-C levels decreased by 13 and 20%, respectively, compared to baseline. Adverse effects were similar in both groups, and the XZK appeared to be well tolerated. A substudy of elderly hypertensive patients in the same CCSPS cohort found that monacolin K was effective in lowering the rates of coronary events and death from CHD compared with placebo [190].

Several clinical trials have shown RYR to be safe, effective, and well tolerated both alone or in combination with other nutraceuticals; however, the studies are small and of short duration [191]. Even if RYR is perceived as a “natural” product providing fewer side effects, it should be taken into account that monacolin K is identical to lovastatin and therefore may present an increased risk of muscular and other side effects especially in patients with a history of SAM. Myopathy, hepatoxicity, and rhabdomyolysis have all been reported in patients taking RYR, as one would expect from any statin therapy [192]. For this reason, RYR should be taken under the guidance of a physician who will closely monitor its efficacy, safety, and tolerability [193]. In the USA, RYR has been used as an alternative to statin therapy in treating patients with mild to moderate hypercholesterolemia, especially among patients who might be intolerant to standard therapy due to statin-associated myalgia (SAM). For this reason, the FDA has prohibited the sale of all RYR products containing monacolin K, because it is considered an unapproved drug; however, many RYR supplements are still on the market.

#### 3.6.4. Allicin

Garlic (*Allium sativum*) has been used as a spice, food, and medicine for over 5000 years and is one of the earliest documented herbs utilized for the maintenance of health (as a diuretic and for the immune system and gastrointestinal health) and for treatment of disease, including circulatory disorders and infections [194]. Functional sulfur-containing components described in garlic include alliin, allicin, diallyl sulfide, diallyl disulfide, diallyl trisulfide, ajoene, and S-allylcysteine. Allicin is a thiosulfinate and in nature is produced after damage of the plant tissue by an enzymatic reaction [195].

Aged garlic extract in cell culture prevented endothelial cell dysfunctions caused by oxidative stress by increasing cellular concentrations of thiol antioxidants, such as cysteine and glutathione (GSH) [196]. A correlation between low red blood cell glutathione (GSH), which plays important roles in cellular redox status and signaling, and increased plasma homocysteine (HCy) has been linked to an increased incidence of hypertension [197]; in an animal model of hyperhomocysteinemia, induced by a severely folate-depleted diet in rats, aged garlic extract decreased plasma HCy concentrations by 30% [198]. The potential effect of garlic on HCy levels has been reported in a small clinical trial of atherosclerosis patients randomized to aged garlic extract (*P* = 0.08) [199]. Garlic has been also shown to have blood pressure- (BP-) lowering properties in hypertensive patients [200]: allicin, decomposes rapidly to its degradation products which results in the release of hydrogen sulfide (H_2_S) [201], a potent gaseous signaling molecule which lowers blood pressure (BP) by the relaxation of smooth muscle cells surrounding the blood vessel [194]. The H_2_S-dependent BP-reducing effect is thought to be primarily mediated through sulfhydration of ATP-sensitive potassium (K_ATP_) channels, which in turn leads to voltage-sensitive channel opening and relaxation of vascular smooth muscle cells [202]. However, other potassium channels may also be affected by H_2_S and additional mechanisms have been suggested in determining the opening/closing of K^+^ channels, including a possible cooperation between H_2_S and NO [202]. In CVD models, the administration of H_2_S prevents myocardial injury and dysfunction [203] and aged garlic extract was shown to normalize NO output from endothelial cells by preventing the decline of BH4 levels [194]. In addition, garlic, due to the high content of polysulfides, may help in providing the nutrients needed for maintaining optimum redox balances for several eNOS-dependent signaling pathways important in vascular relaxation [194]. Additionally, allicin is able to suppress cholesterol biosynthesis [204–206] and platelet aggregation. So far, five trials have demonstrated a strong effect of garlic on inhibition of platelet aggregation, whereas one trial reported no effect [207].

#### 3.6.5. Curcuminoids

Curcuminoids, extracted from the rhizomes of *Curcuma longa*, are naturally occurring polyphenols used for centuries in indigenous medicine to treat various diseases, such as common colds, arthritis, diarrhea, and upper respiratory disorders. The curcuminoids best characterized are curcumin, demethoxycurcumin (DMC), and bisdemethoxycurcumin (BDMC), all belonging to the diarylheptanoid family [208]. A lot of evidence suggests that curcuminoids have a diverse range of molecular targets; curcumin, the main component of curcuma extract, has several biological and pharmacological properties including anti-inflammatory, antioxidant, antithrombotic, antiatherosclerotic, anticonvulsant, and anticancer properties and cardio- and neuroprotective activities (reviewed in [209]).

Curcuminoid treatment improved glycemic factors, hepatic function, and serum cortisol levels in subjects with overweight and impaired fasting glucose in a randomized double-blind placebo-controlled trial involving 80 overweight subjects [210]. Among curcuminoids, curcumin is the best characterized and studied; its antioxidant and anti-inflammatory properties are therefore considered a multifunction phytochemical that can interact with multiple molecular targets, modulating cell growth, inflammation, and apoptosis signaling pathways (reviewed in [211]). Firstly studied for its beneficial properties in the gastrointestinal tract, it has been observed that curcumin has a beneficial role in chronic conditions such as intestinal dysmotility disorders and in the prevention and maintenance of remission of intestinal bowel disease (IBD), requiring long-time treatment [212]. Moreover, curcumin seems to show protective properties from metabolic syndrome, decreasing insulin resistance, obesity, hypertriglyceridemia, and hypertension and preventive properties from complications. It has been evidenced that curcumin possesses hypolipidemic effects, which together with its antioxidant and anti-inflammatory activities can contribute to reducing the incidence of atherosclerosis [213]. The remarkable antioxidant capacity of curcumin reduces lipid peroxidation and the generation of oxLDL and, consequently, reduces the inflammatory response and the progression of atherosclerosis [213].

Recently, it has been observed that curcumin can inhibit hypoxia-inducible factor 1*α* (HIF-1*α*) thus repressing the total cholesterol and lipid level in macrophage under hypoxic condition [214]. The benefit of curcumin in patients at risk of atherosclerosis has also been described; after 6 months of curcumin dietary supplementation, patients with type 2 diabetes had lower pulse wave velocity which improved the metabolic profile [215]. Furthermore, the use of curcumin for 8 weeks improved flow-mediated dilatation in 32 postmenopausal women [216]. A major limitation to using curcuminoids as a nutraceutical is its poor bioavailability, owing to inadequate absorption in the gut and as it is rapidly broken down and quickly excreted from the body. Several strategies are being pursued in an attempt to increase their bioavailability, including the use of liposomal curcumin, nanoparticles, and a curcumin phospholipid complex.

## 4. Notch Signaling Modulation by a Nutraceutical Approach

The Notch signaling is a highly conserved short-intercellular communication system deeply investigated for the possible role as a novel therapeutic target in cancer [217], which is becoming more and more recognized as a key player in the maintenance of vascular homeostasis. In the next paragraphs, we will discuss the basics of this pathway, the role played by its dysregulation in atherosclerosis and what is currently known about the effects of nutraceuticals on Notch.

### 4.1. The Basics of Notch Signaling

**Figure 2:**
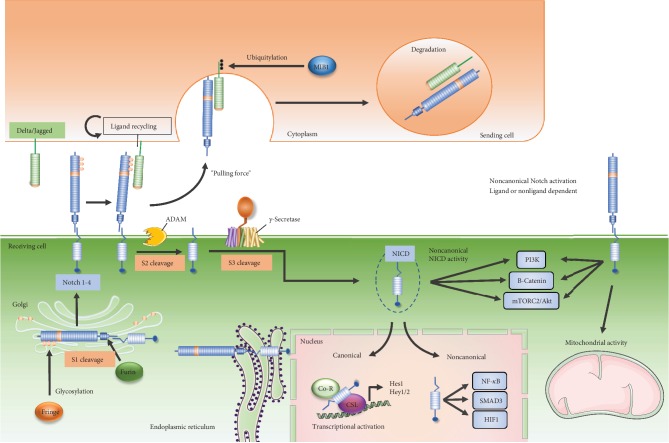
Canonical and noncanonical Notch signaling pathway. In the canonical Notch pathway, precursor of Notch receptors undergoes Furin-mediated cleavage (S1) in the Golgi apparatus, which is necessary to form the functional heterodimeric receptor. Upon Notch glycosylation by the Fringe family of glycosyltransferases, the Notch receptor translocates to the plasma membrane, where it interacts with a Delta/Jagged ligand, present on the surface of an adjacent cell. Notch signaling is activated when the ligand, bound to the receptor, is ubiquitylated by MIB1, an event that generates the mechanical force necessary for exposing the second cleavage site of Notch receptors. This event leads ADAM to perform the second cleavage (S2). The third cleavage (S3), by the *γ*-secretase complex, promotes the release of the intracellular domain of the receptor (NICD). NICD translocates into the nucleus where it promotes the transcription of canonical Notch target genes, such as Hey1 and 2 and HES1. The noncanonical Notch signaling pathway may be *γ*-secretase dependent or independent. This later may also occur either in the presence or in the absence of its ligand. Noncanonical Notch signaling is also independent of CSL, and it is mediated by the interaction with PI3K, mTORC2, AKT, Wnt, NF-*κ*B, YY1, or HIF-1*α* pathways at either the cytoplasmic and/or nuclear levels.

In mammals, there are four highly homologous receptors (Notch-1-4) and five ligands belonging to Delta-like (Dll-1, 3, and 4) or Jagged (Jagged-1 and 2) families. Notch receptors are synthesized as single-chain precursors and are cleaved by Furin (S1 cleavage) into an extracellular domain (NECD, rich in epidermal growth factor- (EGF-) like repeats) and a transmembrane subunit in the Golgi apparatus, generating the functional heterodimeric receptor, linked by Ca^2+^-dependent noncovalent bonds. Here, the EGF-like domains can be modified by the adding of O-fucose glycans by the Glycosil transferase Fringe [218], thus determining which ligands can be bound by the Notch receptors. Similarly, the ligands are also single-pass type I transmembrane proteins and present an extracellular domain formed by EGF-like repeats [219]. The canonical activation of Notch signaling arises from the interaction between the Notch receptors and their ligands on adjacent cells (Figure 2).

Accordingly to the “pulling force” theory, the Notch signaling is activated when the E3 ubiquitin-protein ligase (MIB1) modifies the Notch ligands, when bound to NECD, allowing the ligand endocytosis and generating the mechanical force necessary for exposing the second cleavage site of Notch receptors, thus driving the successive proteolytic cleavages mediated by A disintegrin and metalloprotease (ADAM) surface protease (S2 cleavage) [220]. A second intramembranous cut by *γ*-secretase (S3 cleavage) mediates the release of the Notch intracellular domain (NICD), the active form of the receptor. NICD translocates into the nucleus where it binds the transcription factor CSL (CBF-1, suppressor of Hairless and Lag-1) also known as RBP-J*κ* (recombinant signal-binding protein 1 for J*κ*) transcription factor, thus promoting the transcription of Notch target genes. The most characterized direct Notch target genes are the negative regulator of transcription and belong to the Hairy and Enhancer of Split (HES) and Hairy and Enhancer of Split with YRPW motif (HEY) gene families [221] although several other targets have been described [222]. Under pathological conditions, like cancer and activation of the immune system, Notch signaling can also act in a RPB-J*κ*-independent manner (“noncanonical” fashion) signaling [223]. In the noncanonical pathway, NICD interacts with other transcription factors, such as SMAD3 (small mother against decapentaplegic 3), YY1 (Yin Yang 1), HIF-1*α*, and NF-*κ*B (nuclear factor kappa-light-chain-enhancer of activated B cells), into the nucleus, whereas, in the cytoplasm, this signaling may occur via the uncleaved Notch receptor, still bound to membrane, or via the NICD, through interaction with PI3K (phosphoinositide 3-kinase)/Akt/Wnt/*β*-catenin pathways [224]. Recently, it has been also reported that Notch, by interacting with PTEN-induced kinase 1 (PINK1), can activate the mTORC2/Akt pathway, thus influencing the mitochondrial function and cell survival [225]. Posttranslational modifications (methylation, hydroxylation, acetylation, ubiquitylation, and phosphorylation) and the interplay with other signaling pathways, such as NF-*κ*B, estrogen receptor- (ER-) alpha, G protein-coupled ER (GPER), ErbB2, and vascular endothelium growth factor receptors (VEGFRs) [226–231], increase the complexity of this signaling pathways which, in a cell type- and context-dependent manner, can influence a plethora of biological processes.

### 4.2. Notch in the Endothelium

A large number of *in vitro* and *in vivo* studies have convincingly established the critical role of Notch signaling during development of the vascular system in which Notch is indispensable for a correct arteriovenous specification [232] and its malfunctioning has been correlated to vascular abnormalities, not compatible with life. Briefly, Notch-1 homozygous mutant and Notch-1/Notch-4 double homozygous mutant embryos displayed severe defects in angiogenic vascular remodeling [5, 233, 234]. Similarly, expression of active Notch-4 in vasculature leads to an anomalous vessel structure and embryonic lethality at embryonic day 10 (E10) [235]. Furthermore, the vascular defects of Notch transcriptional regulators (RBP-J*κ* or Mib1) on homozygous mutant mice embryos are similar to defects developed by mutant embryos for Notch receptors and died prior to E11.5 [236, 237]. Likewise, Hey1 or Hey2 and Dll-4 or Jagged-1 mutant mice exhibit defects in their vasculature during embryogenesis and died from massive hemorrhage [238–241].

Given the pivotal role played by Notch signaling in vascular embryogenesis, it is not surprising that Notch is also essential in maintaining the homeostasis of the adult vasculature. In ECs, all Notch receptors, Dll-1 and 4 and Jagged-1 and 2 ligands, are expressed [242] and it is well-known that Notch, by intricate crosstalk with VEGF-A, controls arterial angiogenesis, also under proinflammatory conditions [243], by affecting the balance between tip cells and stalk cell [244].

During the last decade, Notch has been under the spotlight of the atherosclerosis research field since, except for a few studies [116, 245–247], numerous studies demonstrated that Notch may counteract endothelial dysfunction and atherosclerotic plaque development. Quillard et al. provided the first *in vitro* evidence that proinflammatory conditions (i.e., TNF-*α*) dysregulate Notch signaling leading to increased levels of ICAM-1 and VCAM-1 and to NF-*κ*B-mediated apoptosis [248, 249]. These data were corroborated by Briot et al. showing that in human aortic ECs, siRNA-mediated reduction of Notch-1 is sufficient to increase the expression of inflammatory markers and adhesion molecules and that treatment of ECs with oxidized lipids and proinflammatory cytokines (TNF-*α* and interleukin-1-beta (IL1*β*)) decreased Notch-1 expression [250]. In agreement with this study, we have recently demonstrated that 17*β*-estradiol is able to limit TNF-*α*-induced apoptosis in ECs by activating Notch-1 [226]. In support of the protective role of Notch in the endothelium, Wang and collaborators reported that in bone marrow ECs, RBP-J*κ* inhibits miR-155/NF-*κ*B axis activation [251], data being reinforced by the Nus et al. study which showed that RBP-J*κ* heterozygous inactivation results in aortic valve calcification under dyslipidemic condition [252]. Similarly, in human-induced pluripotent stem cell- (iPSC-) derived ECs, Notch-1 haploinsufficiency interferes with EC response to shear stress, causing the unlock of proosteogenic and inflammatory networks [253]. The interplay between Notch and shear stress is an emerging relevant topic in the context of vascular biology [6]. Diverse Notch signaling components in the endothelium respond to shear stress [254], and Notch-1 is essential for preserving EC tight junctions and their normal transcriptional/epigenetic response to shear stress [253, 255]. Polacheck et al. suggested that Notch-1 may be activated by shear stress through a Dll-4-dependent mechanism, triggering a noncanonical Notch pathway [256]. Schober et al. showed instead that in aorta regions exposed to turbulent flow, thus prone to plaque formation, disturbed shear stress may lead to expression of the Notch inhibitor Delta-like 1 homolog (Dlk1), through the downregulation of miR126-5p, leading to reduced expression of *HES5*, a Notch-1 target gene essential for restoring the dyslipidemia-injured endothelium [257]. Noteworthy, we have recently demonstrated that heart rate reduction by ivabradine treatment induces an atheroprotective gene profile and *HES5* expression in the aortic arch endothelium of apolipoprotein E-deficient (ApoE^−/−^) mice, which was linked to maintenance of endothelial integrity and reduction in the plaque area in their aortic root [258].

### 4.3. Notch in Vascular Smooth Muscle Cells

The correct morphology and functionality of VSMCs are also indispensable for guaranteeing the stability and function of adult vasculature, and in the first stages of atherosclerosis, VSMCs, switching from a contractile/quiescent to a secretory/inflammatory/migratory state, play a role in plaque formation. Many studies suggest that Notch is necessary for maintaining VSMCs in a contractile/quiescent phenotype. In rat VSMCs, the IL-1*β*-induced secretory/migrating phenotype is blunted by Notch-3 overexpression and enhanced by treatment with DAPT, a *γ*-secretase inhibitor [259] but, contrary to this finding, DAPT also seems to prevent SMC migration and proliferation induced by AngII [260].

We recently demonstrated that cholesterol-induced VSMC transdifferentiation is associated with reduced levels of Jag1 and Hey2 and increased levels of Dll-4 mRNAs [261]. In human aortic VSMCs, Notch-3 also promotes transcription of prosurvival genes, which resulted to be significantly decreased in the aortas of Notch-3^−/−^ mice [262]. Similarly, Notch-3 knockdown by RNA interference caused VSMC to have higher proliferation, migration, and apoptosis rates, with a concomitant abnormal morphology configuration [263]. Consistently, Ragot et al. showed that the loss of the Notch-3-RBP-J*κ* pathway in VSMCs led to cardiac vasculature alterations in response to AngII-induced hypertension, thus to the development of cardiac hypertrophy [264]. Reduced expression of Notch-1 and Dll-4 has been also found in the aortic wall of patients with abdominal aortic aneurysm, which was correlated to decreased VSMC content in the vessels [265]. Chen and collaborators found that Notch-1 repression is required for miR-34a-mediated VSMC proliferation and migration [266]. Redmond et al. also reported that Notch-1 activation may guide the neointimal formation and VSMC proliferation in the carotid artery ligation mouse model, phenomenon prevented by Notch-1 siRNA injected following carotid ligation [267]. In conclusion, based on the existing data, which seem to indicate an opposite effect of Notch-1 and Notch-3 in controlling VSMC activity and phenotype, the role played by Notch in this context still needs to be clarified.

### 4.4. Notch in Macrophages

During atherosclerotic plaque formation, intraintima macrophages respond to extracellular and intracellular signals which regulate their phenotypes, resulting in high levels of heterogeneity and plasticity among macrophage subtypes [268]. The “classical” model of macrophage activation indicates that the predominant phenotypes are characterized by a proinflammatory M1 and alternative M2 profiles [268]. Many studies *in vitro* clearly indicate that the Dll-4/Notch-1 axis is involved in promoting a M1 phenotype in macrophages [269–272]. Outtz et al. demonstrated that macrophages from Notch-1^+/-^ mice displayed decreased LPS/IFN*γ*-mediated induction of proinflammatory IL-6, IL-12, and TNF-*α* compared with wild-type mice [273]. These data have been confirmed by Xu and collaborators that showed in macrophages isolated from Notch-1^−/−^ mice a decreased basal and LPS-induced NF-*κ*B and HIF-1*α* activation, indicative that induction of the M1 pathway is dependent on Notch signaling [274]. Defects in NF-*κ*B p50 nuclear localization were observed in DAPT-treated macrophages and in RBP-J*κ*-deficent macrophages, indicative of crosstalk between Notch and NF-*κ*B pathways [275]. Lastly, DAPT treatment during MI diminished the number of macrophages in the infarcted area and significantly increased the M2 macrophage polarization [276], data resembling those obtained by Singla et al. that confirmed reduction of the proinflammatory M1 phenotype following monocyte treatment with DAPT or Notch-1 siRNA [277].

Pabois et al., by using an EC/monocyte coculture system, demonstrated that endothelial Dll-4 induces M1 polarization [278], and consistently, Koga et al. showed that Dll-4 antibody administration resulted in reduced vein graft lesion development in LDLr^−/−^ mice and concomitantly decreased macrophage accumulation and expression of proinflammatory M1 genes [279]. Recently, the Pagie et al. study showed that Dll-4, other than promoting LPS/IFN*γ*-mediated M1 polarization, interferes with IL-4-mediated M2 macrophage polarization [280]. In elucidating the mechanism underlying the Notch signaling-mediated M1/M2 polarization, Lin et al. suggested that activation of Notch signaling might downregulate, through HES family corepressors, the signal regulatory protein *α* (SIRP*α*), a M2 phenotype inducer, which conversely resulted to be upregulated in RBP-J*κ*-deficient bone marrow-derived macrophages [281]. Consistently, bone marrow-derived macrophages from RBP-J*κ*^−/−^ mice displayed, under LPS/IFN*γ* stimulation, a M2 phenotype (reduced expression of TNF-*α*, IL-6, and inducible-nitric oxide synthase (iNOS)), which can be reversed by transfection with miR-148a-3p mimic, a Notch-1-target miRNA which promotes M1 polarization [282]. Similarly, Miranda and collaborators, in an attempt to identify the link between the macrophage subtype and the resistance to insulin in HFD-induced obesity mouse models, found that miR-30, targeting Dll-4, is associated with obesity-induced inflammation and proinflammatory cytokine production in adipose tissue macrophages isolated from visceral fat of obese mice [283]. Specifically, they demonstrated that miR-30 inhibition is sufficient to promote the Dll-4/Notch-1 axis and proinflammatory cytokine (TNF-*α* and CCL2) production, this later is blunted by using anti-Dll-4 antibody. Conversely, lentiviral overexpression of miR-30 in RAW264.7 cells resulted in reduced M1 polarization and TNF-*α*/CCL2 production [283]. Taken together, with the previously published studies [269], these studies indicate that Dll-4/Notch-1 assume in macrophage a central function in determining the balance of M1/M2 subpopulations; thus, it could represent a valid anti-inflammatory target for limiting excessive activation of proinflammatory programs during atherosclerosis onset.

### 4.5. Nutraceuticals Acting through the Notch Pathway

Several authors reported that natural extracts can be used in cancer prevention; few of them focused the studies on Notch signaling, obtaining interesting results. EGCG is able to inhibit Notch signaling in BALB/c nude mice, previously injected with cancer stem cells (CSC), inhibiting tumor formation [284]. Honokiol, a phenolic compound isolated from the bark of *Magnolia officinalis* Rehder (Magnoliaceae), is able to counteract the growth of melanospheres formed by CSC isolated from two melanoma cell lines downregulating Notch-2 receptor, HES1, and cyclin D1 expression [285]. Withaferin-A (WA), a bioactive compound derived from Withania somnifera, inhibits Notch-1 signaling and cell proliferation in three colon cancer cell lines [286]. Two bioactive compounds (tricin and p-coumaric acid) present in leaf extract of *Sasa quelpaertensis* Nakai (Poaceae) are able to inhibit the growth of CSCs deriving from different colon cancer cell lines through Dll-1 and Notch-1 inhibition and downregulation of biomarkers related to tumor vascularization (VEGF and HIF-1*α*) [287]. Curcumin inhibits the activation of Notch-1 and the expression of Jagged-1 as well as HES1 in esophageal cancer cell lines [288]. It has also been shown that curcumin inhibits the expression of Notch-1-specific microRNAs including miR-21and miR-34a and upregulates the cancer suppressor let-7a miRNA. Moreover, Notch downstream genes are overexpressed in pancreatic cancer and curcumin induces apoptosis through reduction of the Notch-1 signaling pathway and downregulation of cyclin D1 and Bcl-xL. Curcumin downregulated the expression of Notch-1 leading to increased apoptosis and cell cycle arrest in hepatic and oral cancer cells, activating NF-*κ*B and its target genes (Bcl-2, cyclin D1, VEGF, and MMP-9) [289]. Curcumin inhibits the DNA-binding ability of NICD in prostate cancer cells [290] and decreases also CSC markers in lymphoma/leukemia cells, at least in part through inhibiting their self-renewal [291]. Resveratrol, a natural phenol present in grape skin with known anticancer effects [292], is able to suppress proliferation and to induce a p53-mediated apoptosis in acute lymphoblastic leukemia cell via inhibition of the Notch signaling pathway [293]. These studies suggest that curcumin treatment is an attractive new strategy for several types of cancers at least in part thanks to its capability to downregulate Notch-1 signaling.

In human epidermal growth factor receptor 2- (HER-2-) positive breast cancer cells, characterized by HER2 gene amplification [294], all-trans retinoic acid (ATRA) inhibited *γ*-secretase and Notch-1 processing, involved in cell migration and proliferation [295]. Oroxylin A, a natural compound extracted from the root of *Scutellaria baicalensis*, inhibited the hypoxia-induced invasion and migration of ER*α*-positive breast cancer cells by suppressing the Notch processing [296]. Alpinetin can suppress the proliferation and invasiveness of gastric stem cells (GSCs) and induce apoptosis through Notch inhibition in glioma stem cells [297]. Cowanin has a strong inhibitory activity of the Notch signaling target proteins, HES1 and HES5, affecting the activity of the *γ*-secretase complex on several cancer cells [298].

Still, little is known about the modulation of Notch signaling by natural bioactive compounds and the consequent control of many features that characterize endothelial dysfunction. EGCG prevents the oxLDL decrease of Jagged-1 and Notch pathway-related proteins (MATH1, HES1, and HES5) and inhibits apoptosis *in vitro* in human vascular ECs and in ApoE^−/−^ mice [276]. Furthermore, EGCG inhibits macrophage accumulation and inflammation response in the skin wounds of streptozotocin- (STZ-) induced DM mice at least in part through Notch signaling modulation [299]. DHA, an omega-3 fatty acid, increases Notch-3 and HES1 transcription and enhances *γ*-secretase complex activity, thus reducing fibrinolytic/MMP activity in transdifferentiated VSMCs toward a migrative/proliferative phenotype [300]. Norisoboldine, an alkaloid compound isolated from *Radix Linderae*, suppresses synovial angiogenesis, thanks to its inhibition of VEGF-induced endothelial cell migration via a cAMP-PKA-NF-*κ*B/Notch-1 signaling pathway [301]. Both in rat aortic subintimal macrophages and in *in vitro*-differentiated macrophage cell diosgenin, a phytosteroid sapogenin extracted from the tubers of *Dioscorea* wild yam suppresses the nuclear translocation of NICD [302]. Resveratrol can attenuate neointimal VSMC hyperplasia in rats, following balloon injury, through a mechanism that involves inhibition of the Notch signaling [303]. Activation of Notch signaling reduces myocardial ischemia reperfusion injury (MI/RI), by activating key components of survival pathways, namely, PI3K-Akt, NOS, and mitochondrial K^+^-ATP (mitoKATP) channels [231]; BBR, polydatin, and 2,3,5,4′-tetrahydroxystilbene-2-O-*β*-D-glucoside upregulate Notch-1/HES1 signaling attenuating myocardial apoptosis both in cultured cardiomyocytes and in rats subjected to MI/RI [304–306].

Nutraceuticals have been studied also in the context of age-related diseases [307] and few studies reported an involvement of Notch in the effect of natural extract treatment on Alzheimer disease. Dihydroergocristine (DHEC), a component of ergoloid mesylates approved by the FDA for the treatment of hypertension and dementia, suppresses the production of A*β* peptides by inhibiting the *γ*-secretase complex in neurons [308]. Also, (20S)-Rg3, a triterpene natural compound known as ginsenoside and one compound (SPI-014) isolated from *Actaea racemosa* reduced A*β* peptide levels in neurons *in vitro* and in a mouse model of Alzheimer disease, at least in part decreasing the association of presenilin 1 (PS1) fragments with catalytic components of the *γ*-secretase complex localized in lipid rafts [309].

Indeed, the modulation of Notch signaling in several age-related diseases is an emerging approach in clinical practice and the correct use of plants could help to decrease the incidence as well as the healthcare costs of these pathologies.

## 5. Conclusions

The increased understanding of how diet affects disease, together with high healthcare costs and the aging population, has generated interest in food as a tool for disease prevention and health enhancement. There is growing evidence that components of food may play an integral role in the link between food and health; thus, a diet, mainly based on plant and plant-derived nutraceuticals, can contribute to reduce health care costs, while supporting economic development in rural communities.

Preclinical investigations have consistently shown that bioactive compounds, present in plants and certain foods, inhibit those biological processes linked to atherosclerosis onset. In the clinical settings, while a large number of studies have shown a close association between an imbalanced diet, with low consumption of fruits, vegetables, fibers, vitamins, and fish, and the increase risk of incidence of CVDs, other studies have failed to show results in primary, or secondary, prevention of atherosclerosis-related coronary artery disease, stroke, and heart failure trough consumption of nutraceuticals. This suggests that there is still much left to be understood about the stability of these compounds *in vivo*, the best route and dose of administration, and differences in response related to gender and age. Moreover, more investigations are needed to fully understand how nutraceuticals' effect varies in the presence of those medications commonly used by the subjects involved in this type of studies. This gap could be filled by a wider characterization of the molecular pathways regulated by these compounds. The Notch signaling is a well-known master regulator of embryogenesis and postnatal maintenance of self-renewing tissues, and its detrimental role when trying to achieve cancer cell apoptosis is unquestioned. To date, *γ*-secretase inhibitors (GSIs) are the most studied Notch-inhibiting agents which increase response to chemotherapy [310]. Furthermore, the modulation of Notch signaling by nutraceuticals to interfere with cancer progression (Table 2) is a research field that has been steadily growing in the last ten years.

As discussed in this review, the implications of Notch in atherogenesis are wide ranging, from protection against endothelial dysfunction to the modulation of VSMCs and macrophage phenotypes, but a detailed analysis of the signaling upstream and downstream of Notch, in each cell context, has not been performed yet. Additionally, due to the distinct roles played in the above cited cell type (anti-inflammatory in endothelial cells vs proinflammatory in macrophages), targeting Notch in the context of atherosclerosis could result to be particularly challenging. There is a limited number of studies showing that some classes of nutraceutical compounds exert antiatherosclerotic activity, at least partially, through modulation of Notch signaling (Table 2). Given the important role of Notch in atherosclerosis, it could be of interest to investigate the effect on vascular Notch of widely investigated and used nutraceutical compounds, in order to gain a better understanding of their biological effects. Additionally, high-throughput analyses could be applied to investigate the effects on Notch of novel, still uncharacterized plant compounds. Based on the established role of Notch as anticancer therapy and on the emerging role of this pathway in atherosclerosis, it is not implausible to imagine a combination of specific tissue-targeted nutraceutical Notch modulators as a novel therapeutic strategy in this context.

## Figures and Tables

**Table 1:** Summary of cardiovascular benefits of major nutraceuticals in human studies.

Nutraceutical	Study name	Study type	Number of participants/studies analyzed	Duration	Intervention	Summary of findings	References
Olive oil	NUTRAOLEUM	Clinical trial	58	5 months	30 mL/d of three virgin olive oils (VOOs): (1) a VOO (124 ppm of phenolic compounds and 86 ppm of triterpenes), (2) an optimized VOO (OVOO) (490 ppm of phenolic compounds and 86 ppm of triterpenes), and (3) a functional olive oil (FOO) high in phenolic compounds (487 ppm) and enriched with triterpenes (389 ppm)	Improved plasma HDL levels Decreased the level of systemic ET-1	[35]
	VOHF	Clinical trial	33	3 weeks	VOO (80 mg·kg^−1^), FVOO (500 mg·kg^−1^), and FVOO enriched with phenolic compounds from thyme FVOOT (500 mg·kg^−1^; 1 : 1)	Enhanced HDL content Increased endogenous antioxidant enzymes Reduced DNA oxidation level Increased fecal microbial metabolic activity Ameliorated endothelial function	[36, 37]

n-3 PUFAs	DART	Clinical trial	2.033	6 months	Advised to eat about 300 g/week of oily fish or fish oil supplements giving an equivalent amount of n-3 PUFAs	29% reduction in all-cause mortality	[311]
	GISSI-Prevenzione	Clinical trial	11.324	3.5 years	Supplements of n-3 PUFA (1 g/d), vitamin E (300 mg/d), both, or none	20% reduction for total deaths 30% reduction for cardiovascular death 45% reduction for sudden deaths	[52]
	JELIS	Clinical trial	18.645	5 years	EPA (1800 mg/d) with statin or statin	19% reduction in major coronary events	[51]
		Meta-analysis	7.951			Reduced overall mortality and sudden death Reduced mortality due to MI	[2]
		Meta-analysis	77.917			No significant associations with CHD events and death No significant associations with nonfatal MI	[56]
	Omega-FMD	Clinical trial	74	3 months	Supplements of n-3 PUFA (2 g/d) or placebo	No improvement of endothelial function indices	[57]
	ASCEND	Clinical trial	15,480	7.4 years	Supplements of n-3 PUFA (1 g/d) or placebo	No reduction in the rates of nonfatal serious adverse events	[58]
	REDUCE-IT	Clinical trial	19.212	4.9 years	Supplements of icosapent ethyl (4 g/d) or placebo	25% reduction in primary composite cardiovascular endpoint 26% reduction in secondary composite cardiovascular endpoint	[60]

Flavonoids	Zutphen Elderly Study	Prospective cohort study	805	5 years		Reduced risk of CHD mortality Reduced incidence of MI	[99]
	Rotterdam Study	Prospective cohort study	4807	5.6 years		reduced incidence of MI	[100]
	The Caerphilly study	Prospective cohort study	1900	14 years		No change in incidence of ischemic heart disease	[101]
	The Health Professionals Study	Prospective cohort study	45589	2 years		No association between tea consumption and CVD	[102]
	FLAVO	Clinical trial	37	4 weeks	(-)-epicatechin (100 mg/d), quercetin-3-glucoside(160 mg/d), or placebo	Only (-)-epicatechin improved endothelial function and reduced inflammation	[103]
SCFAs		Umbrella meta-analysis	31 (meta-analysis)			7-24% reduction in CHD and stroke 17-28% reduction in overall morbidity and mortality	[108]
		Meta-analysis	752,848	12.4 years		23% reduction in CVD mortality	[109]

Vitamins	ASAP	Clinical trial	520	3 years	d-Alpha-tocopherol (182 mg/d), slow-release vitamin C (500 mg/d), both, or placebo	Delayed progression of atherosclerosis	[153, 154]
	Women's Health Study	Clinical trial	39.876	10.1 years	Natural-source vitamin E (600 IU) on alternate days	Reduced cardiovascular mortality in healthy women	[155]
	MRC/BHF	Clinical trial	20.536	5 years	Vitamin supplementation (vitamin E, 600 mg/d; vitamin C, 250 mg/d; *β*-carotene, 20 mg/d)	No significant reduction in the incidence of cardiovascular events and CVD-related mortality	[156]
	GISSI-Prevenzione	Clinical trial	11.324	3.5 years	Supplements of n-3 PUFA (1 g/d), vitamin E (300 mg/d), both, or none		[52]
	VEAPS	Clinical trial	353	3 years	DL alpha-tocopherol (400 IU/d) or placebo		[157]
	HOPE	Clinical trial	9.541	4.5 years	Natural-source vitamin E (400 IU/d) or placebo		[158]
	SU.VI.MAX	Clinical trial	1.162	7.2 ± 0.3 years	Combination of antioxidants (vitamin C, 120 mg/d; vitamin E, 30 mg/d; beta carotene, 6 mg/d; selenium, 100 *μ*g/d; zinc 20 mg/d) or placebo		[159]
		Meta-analysis	51 (trials)			No significant reduction in mortality and cardiovascular risk	[160]
		Meta-analysis	15.871			No significant reduction in mortality and cardiovascular risk	[161]
	CARET	Clinical trial	18.314	6 years	Beta-carotene (30 mg/d) and vitamin A (25000 IU/d) or placebo	26% increase of CVD-related mortality	[162]
	Meta-analysis	Meta-analysis	2,000,000			No prevention of heart attacks, strokes, or cardiovascular death	[164]
						Reduced risk of CHD incidence	

**Table 2:** List of nutraceuticals acting through Notch signaling modulation.

Nutraceutical	Disease	Major findings	Role of Notch	References
Epigallocatechin-3-gallate (EGCG)	Cardiovascular	EGCG inhibits macrophage accumulation and inflammation response in the skin wounds of STZ-induced diabetes mellitus	EGCG reduces expression of Notch-1 and 2 in wound tissues of diabetic mice	[299]
		In RAW 264.7, EGCG limits LPS-mediated release of proinflammatory IL-1*β*	EGCG reduces expression of Notch-1 and 2 and of Notch target gene HES1. EGCG binds Notch-1 and limits its activity	
		In HUVECs, EGCG induces expression of iNOS and eNOS and inhibits oxLDL-mediated apoptosis	EGCG restores the expression of Jagged-1 and of target proteins (MATH1, HES1, and HES5)	[276]
			Jagged-1 is the key effector of EGCG-protective effect against oxLDL-induced endothelial dysfunction	
		EGCG attenuates the HFD-induced accumulation of atherosclerotic plaque in ApoE-deficient mice	EGCG protects ApoE-KO mice from atherosclerosis through the Jagged-1/Notch-1 pathway	
	Cancer	EGCG inhibits the self-renewal capacity of head and neck squamous carcinoma (HNSC) cancer stem cells (CSCs) by suppressing their sphere forming capacity and attenuates the expression of stem cell markers. EGCG augments cisplatin-mediated chemosensitivity	EGCG decreases HNSC CSC traits by inhibiting the Notch-1 pathway	[225]
Norisoboldine	Cardiovascular	Norisoboldine suppresses VEGF-induced HUVEC migration	Norisoboldine induces VEGF-mediated migration through activation of Notch-1	[301]
Docosahexaenoic acid (DHA)	Cardiovascular	DHA significantly decreases VSMC migration/proliferation induced by IL-1*β* as well as fibrinolytic/MMP activity	DHA increases Notch-3 expression and HES1 transcription and enhances *γ*-secretase complex activity	[300]
Diosgenin	Cardiovascular	Diosgenin reduces the HFD-induced atherogenesis in rat aorta	Diosgenin prevents nuclear translocation of NICD in aorta and in differentiated macrophage cells	[302]
Berberine (BBR)	Cardiovascular	BBR significantly improves cardiac function recovery and decreases myocardial apoptosis, infarct size, serum creatine kinase, and lactate dehydrogenase levels in rats following myocardial IRI	Both *in vitro* and *in vivo*, BBR upregulates NICD translocation and HES1 expression	[304]
		In H9C2, BBR attenuates simulated IRI-induced myocardial apoptosis	*In vitro*, antiapoptotic effect of BBR is blocked by Notch-1 or HES1 siRNA	
Polydatin	Cardiovascular	Following myocardial IRI, polydatin preserves cardiac function, ameliorates myocardial oxidative/nitrative stress damage, and reduces myocardial infarct size in STZ-induced diabetic rats	Polydatin exerts cardioprotection against diabetic myocardial IRI by activating myocardial Notch-1/HES1 signaling. DAPT blunts the beneficial effects of polydatin	[305]
2,3,5,4′-Tetrahydroxystilbene-2-O-*β*-D-glucoside (TSG)	Cardiovascular	TSG significantly improves cardiac function and suppresses IRI-induced myocardial apoptosis	Both *in vitro* and *in vivo*, TSG upregulates NICD and HES1 expression	[306]
		In H9C2, TSG pretreatment dose-dependently decreases simulated IRI-induced apoptosis	*In vitro*, antiapoptotic effect of TSG is blocked by DAPT	
Honokiol	Cancer	Honokiol inhibits the growth of melanospheres formed by CSC	*In vitro*, Honokiol downregulates Notch-2, HES1, and cyclin D1 expression	[285]
Withaferin-A	Cancer	In three colon cancer cell lines, Withaferin-A mediates c-Jun-NH(2)-kinase-mediated apoptosis	Withaferin-A inhibits Notch-1 signaling	[286]
Tricin and p-coumaric acid	Cancer	Tricin and p-coumaric acid inhibits the growth of CSCs and VEGF and HIF1*α* expression	Tricin and p-coumaric acid inhibits Dll-1 and Notch-1 expression	[287]
Curcumin	Cancer	Curcumin inhibits hepatocellular cancer cell (HCC) proliferation	Curcumin decreases NICD expression in HCC	[289]
		Curcumin treatment results in a 40% decrease in tumor growth in a nude mouse xenograft model		
		Curcumin inhibits proliferation and colony formation in esophageal cancer cell lines and upregulates expression of let-7a miRNA	Curcumin reduces Notch-1 activation and expression of Jagged-1 and HES1	[288]
			Curcumin reduces expression of Notch-1-specific microRNAs (miR-21 and miR-34a) and upregulates tumor suppressor let-7a miRNA	
		Curcumin decreases markers associated with CSCs in Burkitt lymphoma and acute myeloid leukemia cells	Curcumin reduces expression of Notch-1 and cyclin D1	[93]
Resveratrol	Cancer	Resveratrol increases apoptosis and suppresses proliferation in MOLT-4 acute lymphoblastic leukemia cells	Resveratrol reduces NICD levels in a dose-dependent manner and inhibits the expression of HES1	[293]
	Cardiovascular	Resveratrol inhibits phenotypic switching of neointimal VSMCs after balloon injury in rats	Resveratrol decreases Notch-1, Jagged-1, Hey1, and Hey2 mRNA in balloon-injured arteries at 7 days	[303]
All-trans retinoic acid (ATRA)	Cancer	ATRA exerts a strong antimigratory action in the HER2-positive SKBR3 cell line	ATRA inhibits Notch-1 pathway	[295]
Oroxylin A	Cancer	Oroxylin A inhibits the hypoxia-induced invasion and migration of ER*α*-positive breast cancer cells	Oroxylin A inhibits NICD translocation into the nucleus	[296]
Alpinetin	Cancer	Alpinetin suppresses the proliferation and invasiveness of glioma stem cells (GSCs) and induces their apoptosis	Alpinetin reduces Notch-1 activity. Notch reactivation, by using recombinant Jagged-1, rescues the effect of alpinetin on GSCs	[297]
Cowanin	Cancer	Cowanin shows potent cytotoxicity against human leukemic HPB-ALL cells	Cowanin degrades nicastrin, a component of *γ*-secretase, thus hampering Notch-1 activation	[298]
